# The association between trajectories of risk factors and risk of cardiovascular disease or mortality among patients with diabetes or hypertension: A systematic review

**DOI:** 10.1371/journal.pone.0262885

**Published:** 2022-01-27

**Authors:** Yuan Wang, Eric Yuk Fai Wan, Ivy Lynn Mak, Margaret Kay Ho, Weng Yee Chin, Esther Yee Tak Yu, Cindy Lo Kuen Lam

**Affiliations:** 1 Department of Family Medicine and Primary Care, Li Ka Shing Faculty of Medicine, The University of Hong Kong, Hong Kong SAR, China; 2 Department of Pharmacology and Pharmacy, Li Ka Shing Faculty of Medicine, The University of Hong Kong, Hong Kong SAR, China; 3 Laboratory of Data Discovery for Health (D24H), Hong Kong Science and Technology Park, Sha Tin, Hong Kong SAR, China; Kurume University School of Medicine, JAPAN

## Abstract

**Introduction:**

Cardiometabolic risk factors and renal function are monitored regularly for patients with diabetes mellitus (DM)/ hypertension (HT). In addition to risk factor levels at a single time point, their trajectory (changes over time) can also be differentially related to the risk of cardiovascular diseases (CVD) and mortality. This study aimed to systematically examine the evidence regarding the association between risk factor trajectories and risk of CVD/mortality in patients with DM/HT.

**Method:**

PubMed, MEDLINE, and Embase were searched for articles from January 1963 to April 2021. Inclusion criteria: studies that 1) analyzed trajectories of risk factors including haemoglobin A1c (HbA1c), blood pressure, estimated glomerular filtration rate (eGFR), body mass index (BMI), and blood lipids; 2) were performed in the DM/HT population and, 3) included risk of CVD/mortality as outcomes. Study quality was assessed using the Newcastle-Ottawa quality assessment scale.

**Results:**

A total of 22,099 articles were identified. After screening by title and abstract, 22,027 articles were excluded by irrelevant outcomes, exposure, population, or type of articles. Following full-text screening, 11 articles investigating the trajectories of HbA1c (N = 7), systolic blood pressure (SBP) (N = 3), and eGFR (N = 1) were included for data extraction and analysis. No studies were identified examining the association of BMI or lipid trajectories with CVD/mortality. All included studies were of good quality based on the NOS criteria. In general, stable trajectories within optimal ranges of the risk factors (HbA1c: <7%, SBP: 120-139mmHg, eGFR: >60mL/min/1.73m^2^) had the lowest CVD/mortality risk compared to an increasing HbA1c trajectory (from 8% to 10%), an increasing SBP trajectory (from 120–139 to ≥140mmHg), or a decreasing eGFR trajectory (from 90 to 70mL/min/1.73m^2^).

**Conclusion:**

A relatively stable and well-controlled trajectory for cardiometabolic risk factors was associated with the lowest risk of CVD/mortality. Risk factor trajectories have important clinical implications in addition to single time point measurements. More attention should be given to patients with suboptimal control and those with unstable trends of cardiometabolic risk factors.

## Introduction

Diabetes mellitus (DM) affected 425 million, or 8.8% of adults aged 20–79 years worldwide in 2017 [1]. An estimated 1.13 billion people globally had hypertension (HT) in 2019 [2]. Patients with DM or HT have a higher risk of cardiovascular disease (CVD) [3–5]. Previous studies have identified a significant association between the risk of CVD and mortality and cardiometabolic risk factors including haemoglobin A1c (HbA1c) or fasting glucose [6, 7], blood pressure [8, 9], estimated glomerular filtration rate (eGFR) [10, 11], body mass index (BMI) [12, 13], and lipids [14–16]. However, most studies have only investigated cardiometabolic risk factors measured at a single point or as an average value over a period, which may not completely capture the longitudinal changes in the risk factor of interest. Longitudinal changes in risk factors are associated with CVD and mortality. Luk et al. found that higher HbA1c variability, measured by the standard deviation, was associated with increased risks of CVD among DM patients in Hong Kong. Such an association was independent of the mean HbA1c [17]. Mehlum et al. identified a positive association between blood pressure variability and risk of CVD in HT patients after adjusting for mean blood pressure [18]. Ohlsson et al. tracked BMI changes during puberty in 37,672 Swedish men and detected different CVD risks among groups classified by various BMI trends from age 8 years to age 20 years [19]. A trajectory integrates serial measurements that can reflect longitudinal trends of risk factors, including stable, increasing, decreasing or fluctuating trends. Trajectories can be different even if patients have similar baseline values, which would be missed in studies that only investigated cardiometabolic risk factors by single measurements. It is suggested that the risk of CVD and mortality may be associated with trajectories of cardiometabolic risk factors [20–24].

In recent years, cardiometabolic risk factor trajectories have been investigated by classifying patients into different groups based on their trajectory patterns and looking for associations between the groups and risks of adverse clinical outcomes [25]. Although trajectory analyses are increasingly being applied in clinical research to assess or predict mortality or cardiovascular outcomes [25], the findings in DM and HT populations have not been systematically reviewed. DM and HT patients may have distinct trajectory patterns and a higher risk of CVD and mortality than other populations. Such studies might yield different results due to study design or population differences. This study aimed to systematically review the current evidence on the association between risk of CVD or mortality and the trajectory of cardiometabolic risk factors, including HbA1c, blood pressure, eGFR, BMI, and lipids, in patients with DM or HT.

## Method

### Literature search

This systematic review was conducted following the Preferred Reporting Items for Systematic Reviews and Meta-Analyses (PRISMA) statement [26]. PubMed, MEDLINE, and Embase were searched to find relevant articles dated 1^st^ January 1963 to 9^th^ April 2021. The search strategy consisted of search terms for outcomes (“cardiovascular” OR “coronary disease*” OR “myocardial ischaemia” OR “coronary artery disease” OR “infarct*” OR “stroke*” OR “heart failure*” OR “death*” OR “die” OR “mortalit*” OR “CVD” OR “CHD”), search terms for cardiometabolic risk factors including HbA1c, blood pressure, lipid, eGFR, and BMI (and Medical Subject Heading terms of them), search terms for a trajectory analysis ("trajector*" OR "trend*" OR "longitudinal*" OR "long-term change*" OR "track*" OR "secular trend" OR "progression*" OR “latent class growth model*” OR “latent class growth mixture model*” OR “growth mixture model*” OR “latent growth model*” OR “latent class growth analysis” OR “latent class growth analyses” OR “group based trajectory model*” OR “group based trajectory analysis” OR “group based trajectory analyses” OR “group based model*” OR “latent growth mixture model*” OR “group based trajector*”), and search terms for the study population (“diabet*” OR “hypertensi*”). The detailed search strategy is listed in S1 Table. Additionally, citation searching was conducted on relevant articles obtained from the literature search.

### Study selection and eligibility criteria

Two analysts (Y.W. and M.H.) conducted the study selection based on the following inclusion criteria: 1) longitudinal observational studies that classified trajectories using trajectory clustering models including group-based trajectory models, latent class mixed models, growth mixture models, k-means clustering method or other data-driven trajectory clustering methodologies that discover patterns and classify trajectories wholly based on the dataset, 2) the exposure consisted of measurements of HbA1c, blood pressure, lipids, eGFR, or BMI, and 3) the outcome was CVD (such as coronary heart disease, stroke and heart failure) or mortality.

Studies were excluded if they 1) were not written in English, and 2) included participants aged <18 years.

Two authors (Y.W. and M.H.) screened all identified articles to obtain a list of studies that met the inclusion criteria. Any differences among authors’ lists were resolved by discussion. Another two authors (E.Y.F.W. and I.M.) adjudicated any unresolved articles.

### Data collection and quality assessment

One author (Y.W.) extracted the data onto a structured data collection form. Data collected included the year of publication, country and/or region, exposure, outcome, the statistical approach used for trajectory classification and outcome risk estimation respectively, study population, study design, sample size, mean or median of age, the proportion of males, length of follow up period, mean or median of DM duration, number and nature of identified trajectory groups, the association between identified trajectory groups and risk of outcomes, period of patient inclusion, exposure period for generating trajectories, outcome follow-up period, criterion to measure the goodness of fit of trajectory clustering models, and quality assessment results. All the collected data were checked by another author (M.H.) independently.

The quality of the studies was assessed using the Newcastle-Ottawa quality assessment scale (NOS) [27]. Eight items of the assessment scale were checked for each of the studies. Studies with at least three stars in the selection domain, at least one star in the comparability domain and at least two stars in the outcome/exposure domain were graded to be of good quality based on the NOS criteria. Two authors (Y.W. and M.H.) individually assessed the quality of all the identified studies to reduce potential publication bias in this review.

## Result

### Study selection

**Fig 1:**
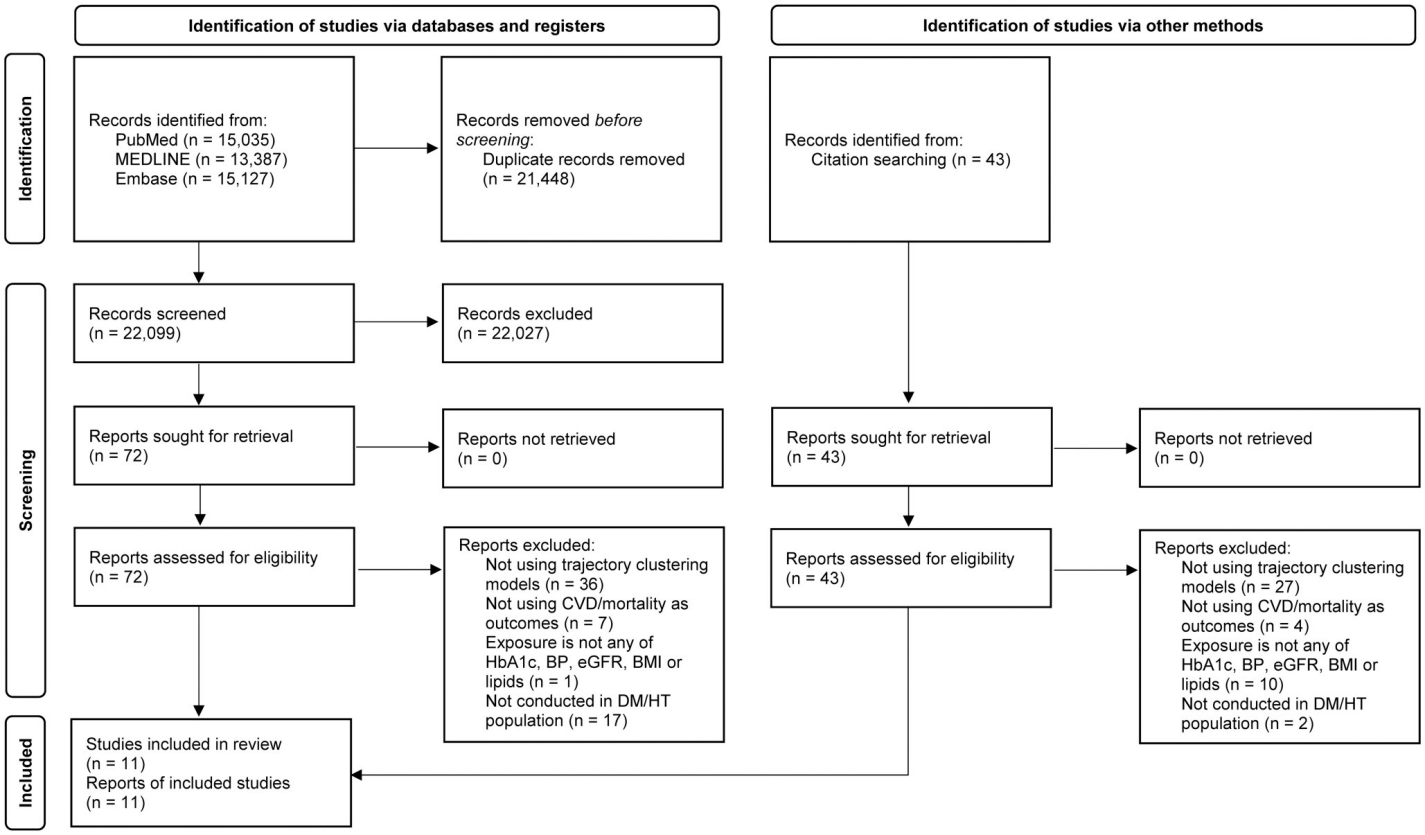
Flow chart for study selection. CVD = cardiovascular disease; HbA1c = haemoglobin A1c; BP = blood pressure; eGFR = estimated glomerular filtration rate; BMI = body mass index; DM = diabetes mellitus; HT = hypertension.

Fig 1 shows the PRISMA flow diagram [26]. A total of 22,099 articles were identified from PubMed, MEDLINE, and Embase after removing duplicates. After screening by title and abstract, 22,027 papers were excluded for irrelevant outcomes, exposure, population, or type of articles, resulting in 72 studies proceeding to full-text review. During full-text review, 61 studies were excluded: 36 for irrelevant study design as trajectory clustering models were not used to classify patients based on their trajectory patterns; eight for irrelevant outcomes or exposures, as there were either no CVD or mortality outcomes (N = 7), or it, did not analyze the trajectory of HbA1c, blood pressure, eGFR, BMI, or lipid trajectories (N = 1); and 17 for having a population that were not DM or HT patients. A further 43 relevant studies were subsequently identified through citation hand-searching, but all were excluded. A total of 11 studies were included for data extraction. All included studies were graded as good quality based on the NOS criteria.

### Study characteristics and results

Characteristics and results of the included studies are summarized in Tables 1 and 2, and S2 Table. Among all 11 included studies, seven investigated the trajectory of HbA1c, three studied the trajectory course of systolic blood pressure (SBP), and one examined eGFR. No studies analyzed BMI or lipids trajectory. There were no studies that analyzed diastolic blood pressure separately without SBP. Ten studies were performed in DM patients, among which eight were restricted to Type 2 DM only while the other two did not distinguish between types of DM. One study was performed on patients with HT.

**Table 1:** Characteristics of identified studies.

Study (Author, Year of publication, Country/region)	Exposure	Outcome	Study population	Study design	Sample size	Age (Mean/ median, years)	Male (%)	Length of follow-up, years	Mean (median) DM duration at baseline, years
Sridharan Raghavan et al., 2020, United States	HbA1c	Mortality	DM diagnosed veterans	Retrospective cohort study	7,780	62	96.3%	2	1.1
Beatriz Hemo et al., 2020, Israel	HbA1c	CVD; Mortality	T2DM patients	Retrospective cohort study	27,724	-	53.7%	8	-
Miyang Luo et al.,2017, Singapore	HbA1c	CVD; Mortality	T2DM patients	Prospective cohort study	5,513	62 (median)	48.6%	9 (stroke); 11 (death)	9
Hsing-Yi Chang et al., 2014, Taiwan	HbA1c	CVD	T2DM patients	Post hoc analysis for RCT	1,091	56	47.9%	4.5	10
Timothy M E Davis et al., 2016, Australia	HbA1c	Mortality	T2DM patients	Prospective cohort study	531	62	54.2%	16	0.6 for group 1;
									3.1 for group 2;
									9.2 for group 3
									(median)
Tomas Karpati et al., 2018, Israel	HbA1c	Mortality	T2DM patients	Retrospective cohort study	60,423	64	47.4%	5	5
Neda Laiteerapong et al., 2016, United States	HbA1c	Mortality	T2DM patients	Retrospective cohort study	25,732	56	53.7%	13.6	Newly diagnosed DM
SanketS. Dhruva et al.l, 2017, United States	SBP	CVD	HT patients	Post hoc analysis for RCT	39,763	67	53.8%	1.5	-
Zhijun Wu et al., 2016, China	SBP	CVD; Mortality	DM patients without HT	Prospective cohort study	3,159	54	81.4%	8	-
Iris Walraven et al., 2015, Netherlands	SBP	Mortality	T2DM patients	Prospective cohort study	5,711	61	50.8%	9	1
Timothy M E Davis et al., 2016, Australia	eGFR	Mortality	T2DM patients	Prospective cohort study	532	62	48.60%	16	3.9

HbA1c = Haemoglobin A1c, SBP = systolic blood pressure, eGFR = estimated glomerular filtration rate, CVD = cardiovascular disease, DM = diabetes mellitus, T2DM = type 2 diabetes mellitus, RCT = randomized controlled trial.

**Table 2:** Statistical models and results in identified studies.

Study (Author, Year of publication, Country/region)	Exposure	Trajectory clustering model	Outcome risk estimation model	Identified trajectory groups	Association between identified trajectory groups and risk of outcomes
Sridharan Raghavan et al., 2020, United States	HbA1c	Joint latent class mixed models	Joint latent class mixed models	A. Stable (around 6.7%)	Increase group has a higher risk of mortality than the stable group
				B. Decline (12% to 8%)	
				C. Increase (8% to 10%)	
Beatriz Hemo et al., 2020, Israel	HbA1c	Latent growth mixed models	Cox proportional hazard models	A. Steady plateau (around 6.7%)	Sharp incline group has a higher risk of mortality and CVD than the steady plateau group
				B. Sharp incline (8.5% to 10%)	
Miyang Luo et al., 2017, Singapore	HbA1c	Latent class growth models	Cox proportional hazard models	A. Low stable (around 7%)	High decrease group has a higher risk of CVD than the low stable group;
				B. Moderate stable (around 8.5%)	
					Moderate increase and high decrease groups have higher risks of mortality than the low stable group
				C. Moderate increase (10% to 11%)	
				D. High decrease (12% to 8%)	
Hsing-Yi Chang et al., 2014, Taiwan	HbA1c	Group-based trajectory models	Cox proportional hazard models	A. Low (around 6.8%)	Intermediate and high groups have higher risks of CVD than the low group
				B. Intermediate (around 8.5%)	
				C. High (around 11%)	
Timothy M E Davis et al., 2016, Australia	HbA1c	Group-based trajectory models	Cox proportional hazard models	A. Low (around 6%)	Group1 (DM duration<1 year): medium group has a higher risk of mortality than the low group;
				B. Medium (around 7.5%)	
					Group2 (DM duration of 1–5 years): high group has a higher risk of mortality than the low group;
				C. High (around 9%)	
					Group3 (DM duration≥5 years): medium and high groups have lower risks of mortality than the low group
Tomas Karpati et al., 2018, Israel	HbA1c	Longitudinal unsupervised trajectory clustering methodology	Chi-square test	A. Stable (around 6.5%)	Descending group has a higher incidence of mortality than the other two groups
				B. Descending (9% to 7%)	
				C. Ascending (7% to 8%)	
Neda Laiteerapong et al., 2016, United States	HbA1c	Latent growth mixture models	Cox proportional hazard models	A. Low stable (around 7.2%)	High decreasing early group has a higher risk of mortality than the low stable group
				B. High decreasing early (11.5% to 8%)	
				C. Moderate increasing late (8% to 11.5%)	
				D. Moderate peaking late (8.5% to 11% (reached at the third year) to 8%)	
				E. Moderate peaking early (8% to 11 (reached at the eighth year) to 8.5%)	
SanketS. Dhruva et al., 2017, United States	SBP	Growth mixture models	Cox proportional hazard models	A. Immediate response (145 to 135mmHg)	Nonimmediate response group has a higher risk of CVD than the immediate response group
				B. Nonimmediate response (150 to (steeply) 160 to (gradually) 150mmHg)	
Zhijun Wu et al., 2016, China	SBP	-	Cox proportional hazard models	A. Stable <120 mmHg	Group F has a higher risk of CVD than group E;
				B. <120 to 120–139	Group A and D have higher risks of mortality than group E
				C. <120 to ≥140	
				D. 120–139 to <120	
				E. Stable 120–139	
				F. 120–139 to ≥140	
Iris Walraven et al., 2015, Netherlands	SBP	Latent class growth models	Cox proportional hazard models	A. Adequate SBP control (around 140mmHg)	Nonresponders group has a lower risk of mortality than the adequate SBP control group
				B. Delayed responders (180 to 140mmHg)	
				C. Insufficient SBP control (150 to (first 3.5 years) 180 to (following 5.5 years) 150mmHg)	
				D. Nonresponder class (150 to 180mmHg)	
Timothy M E Davis et al., 2016, Australia	eGFR	Group-based trajectory models	Cox proportional hazard models	A. Low (within 35-45mL/min/1.73^2^)	Low and High/declining groups have higher risks of mortality than the medium group
				B. Medium (within 60-70mL/min/1.73^2^)	
				C. High (around 80mL/min/1.73^2^)	
				D. High/declining (90 to 70mL/min/1.73^2^)	

HbA1c = Haemoglobin A1c, SBP = systolic blood pressure, eGFR = estimated glomerular filtration rate, CVD = cardiovascular disease.

#### HbA1c

All seven studies were conducted in the US, Israel, Singapore, Taiwan and Australia [28–34]. The length of the follow-up ranged from two to 16 years. The mean DM duration of patients ranged from one to ten years. The mean age of patients ranged from 56 years to 64 years, and the mean proportion of males ranged from 47% to 54%, except for one study on veterans where 96% of the study population were males [34].

**Fig 2:**
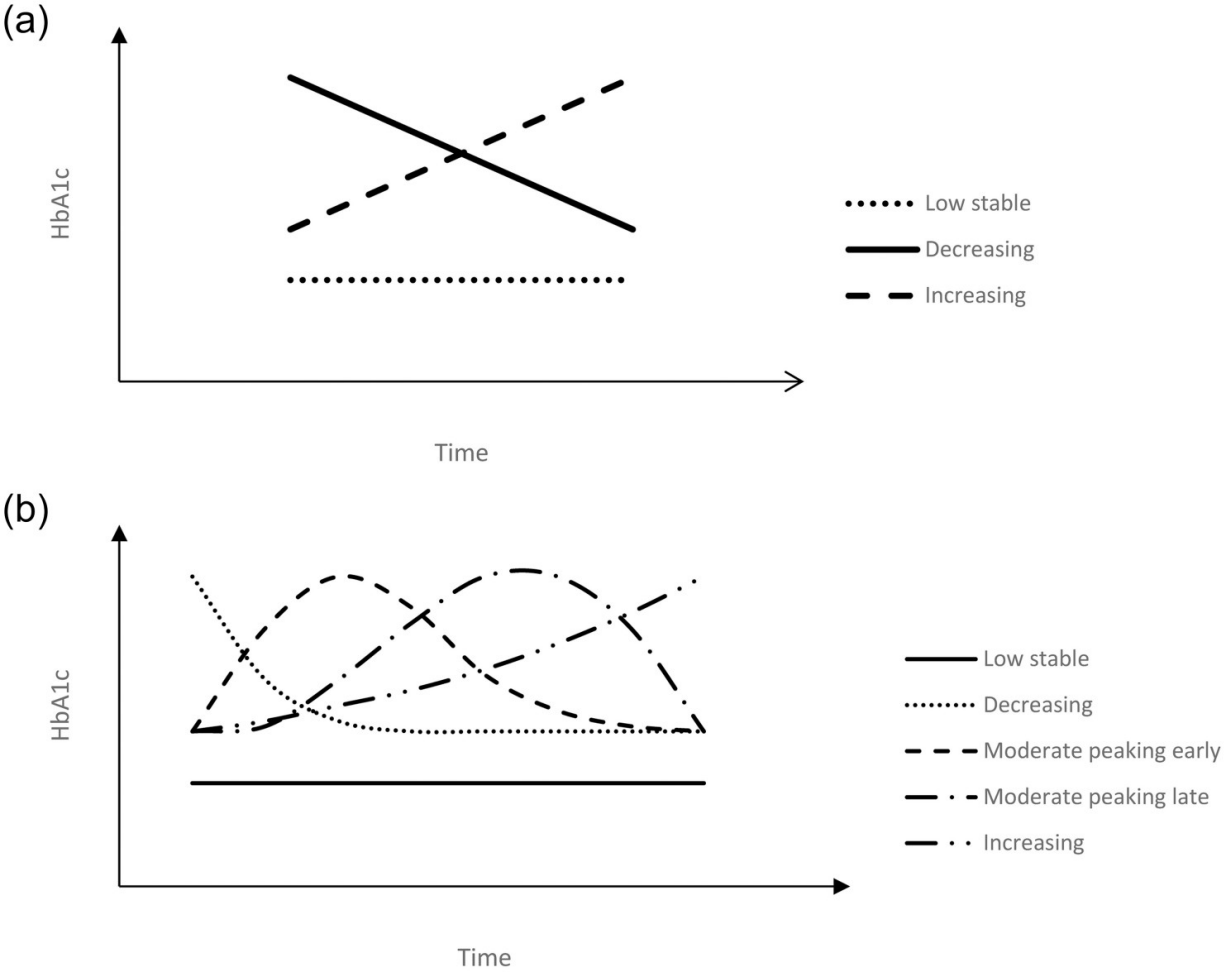
a and b Illustrative representation of HbA1c trajectories.

Four out of seven studies classified HbA1c trajectories into three groups [28, 29, 31, 34]. The other three studies classified HbA1c trajectories into two, four and five groups [30, 32, 33]. Fig 2a illustrates the general patterns of the trajectory groups in three studies: a low stable group (approximately 6.5%, 6.7%, and 7%), an increasing group (from 7% to 8%, from 8% to 10%, and from 10% to 11%), and a decreasing group (from 9% to 7%, from 12% to 8%, and from 12% to 8%), with both the increasing and decreasing group located above the low stable one and intersected with each other [31, 33, 34]. As shown in Fig 2b, one study had five distinct trajectory groups, with a low stable group (around 7.2%), an increasing group (from 8% to 11.5%), a decreasing group (from 11.5% to 8%), a “moderate peaking early” group with moderate initial HbA1c values (around 8.5%) which increased to a high level (around 11%) in the first three years and then decreased gradually (to around 8%) in the following seven years, and a “moderate peaking late” group with moderate initial HbA1c values (around 8%) which increased to a high level (around 11%) gradually in the first seven years and decreased rapidly (to around 8.5%) in the following three years [32]. Two studies had near-parallel trajectory groups, with a low trajectory group (around 6.8% and around 6%), a medium trajectory group (around 8.5% and around 7.5%), and a high trajectory group (around 11% and around 9%) [28, 29]. The study with two trajectory groups had a low stable trajectory group (around 6.7%) and an increasing trajectory group (from 8.5% to 10%) [30].

**Fig 3:**
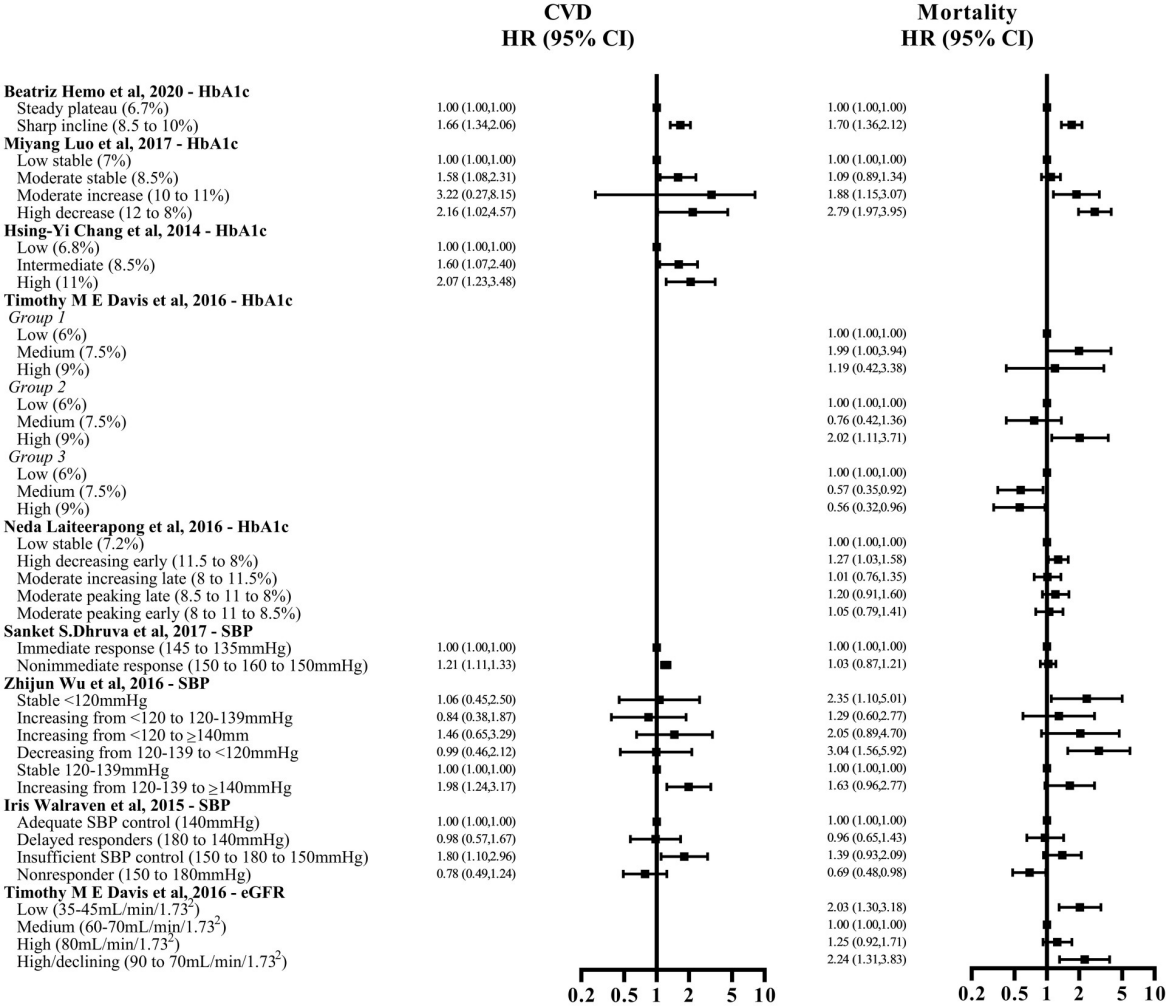
Figure summarizing the hazard ratios reported in identified studies. CVD = cardiovascular disease; HR = hazard ratio; CI = confidence interval; HbA1c = haemoglobin A1c; SBP = systolic blood pressure; eGFR = estimated glomerular filtration rate.

The hazard ratios reported in identified studies were summarized in Fig 3. Patients with an increasing HbA1c trajectory were found to have higher risks for CVD or mortality when compared with patients in the stable HbA1c trajectory group in six of the studies [28–30, 32–34]. One out of the two studies found near-parallel trajectories for patients with a more prolonged diabetes (≥5 years). A low stable trajectory was associated with a higher mortality risk compared with the increasing or decreasing trajectory groups. However, this was not observed among patients with a shorter duration of diabetes (<1 year or 1–4.9 years) [29]. In another study, Karpati et al found that a decreasing HbA1c trajectory (from 9% to 7%) was associated with a higher cumulative incidence of mortality compared with the low stable group (around 7%) and the increasing group (from 7% to 8%) [31].

#### SBP

Three studies (Tables 1 and 2) were conducted based on SBP trajectories. Two were conducted in patients with DM, and one was in patients with HT [35–37]. All patients were recruited from the US, China, and the Netherlands. One study was a post-hoc analysis of a randomized controlled trial (RCT) with a follow-up period of 1.5 years [35]. The other two were cohort studies with a follow-up period of 8–9 years [36, 37]. The mean age of patients ranged from 54 to 67 years, and the mean proportion of male patients ranged from 51% to 81%.

The post-hoc RCT analyses found two trajectory groups, namely the immediate responder and non-immediate responder groups [35]. The immediate responder group had an SBP trajectory decreasing steeply in the first month (from 145 to 135 mmHg) followed by a stable trajectory (around 135 mmHg) in the following five months. The non-immediate responder group had an initial increase in SBP in the first month (from 150 to 160 mmHg), after which SBP began to decrease gradually from 160 to 150 mmHg. The cohort study by Walraven et al identified four trajectory groups, including an adequate SBP control group (stable SBP trajectory around 140mmHg), a delayed responders group (SBP trajectory decreased from 180 to 140 mmHg), an insufficient SBP control group (SBP trajectory increased first from 150 to 180 mmHg and then decreased from 180 to 150 mmHg), and a non-responders group (SBP trajectory increased from 150 to 180 mmHg) [36]. The third study didn’t provide any illustrative figures for the six identified trajectory groups but only described them using the range of SBP values at start time(<120 and 120–139 mmHg) and end time(<120, 120–139, and ≥140 mmHg), with six groups each defined by one of the initial values to one of the end values [37].

Two studies investigated the risk of CVD as an outcome. They found similar results (Fig 3), where an SBP trajectory with an increasing trend was associated with a higher risk of CVD compared with the stable or decreasing trajectories [35, 37]. Conversely, the risk of mortality was higher in the low or decreasing SBP trajectory groups when compared with a higher or increasing SBP trajectory in DM patients, as reported by two studies [36, 37]. One study reported no significant associations between SBP trajectory and risk of mortality in HT patients [35].

#### eGFR

One Australian study (Tables 1 and 2) analyzed the association between eGFR trajectories and mortality risk [38]. It was a cohort study conducted in the DM population recruited from Australia with a follow-up period of 16 years. The mean age was 62 years, and the mean proportion of males was 49%. In this study, eGFR trajectories were classified into four trajectory groups, including a low trajectory group (35–45 mL/min/1.73m^2^), a medium trajectory group (60–70 mL/min/1.73m^2^), a high trajectory group (around 80 mL/min/1.73m^2^), and a high declining trajectory group (from 90 to 70 mL/min/1.73m^2^). The low, medium and high trajectory groups are nearly parallel with a declining rate of 1.6–1.8 mL/min/1.73m^2^ per year, while the high declining has a decline of 4.0 mL/min/1.73m^2^ per year. Patients with a low eGFR trajectory or high declining eGFR trajectory were associated with a higher risk of mortality when compared with patients with a medium eGFR trajectory (Fig 3).

## Discussion

The studies included in this review found similar patterns for the association between risk of CVD or mortality and the trajectory of several cardiometabolic risk factors. HbA1c trajectories with increasing trend, relatively low or decreasing eGFR trajectories, and relatively high SBP trajectories were associated with higher risks of CVD and mortality than a stable trajectory among patients with DM or HT. These results are consistent with previous findings, as a higher CVD risk or mortality is usually associated with higher HbA1c, higher SBP, or lower eGFR [8, 20, 39].

### HbA1c

Studies included in the review found that compared with low, stable HbA1c trajectories, high or increasing trajectories were associated with a higher risk of CVD and mortality in DM patients. This finding supports the positive association between HbA1c and risk of CVD and mortality observed in the UK prospective diabetes study (UKPDS) [40]. Moreover, Nathan et al established a similar association that higher HbA1c was associated with an increased risk of CVD outcomes in the Diabetes Control and Complications Trial (DCCT) [41]. Three out of the seven studies estimated outcome risks adjusting for mean or baseline HbA1c value and still found significant different risks of CVD or mortality between the group with stable HbA1c trajectory and other groups, indicating that the effect of HbA1c trajectory is independent of mean or baseline HbA1c values. The difference in HbA1c variability might explain some of the effects as either increasing or decreasing trajectories would lead to different variability from a stable trajectory [30, 32, 33]. This was consistent with the identified association between HbA1c variability and risk of CVD or mortality in previous studies [17, 42, 43].

Some studies reported conflicting conclusions. Karpati et al showed that a decreasing HbA1c trajectory (from 9% to 7%) was associated with a higher cumulative incidence of mortality compared with stable (around 7%) or increasing (from 7% to 8%) HbA1c trajectories [31]. This inconsistent finding might be explained by the results without adjustments for patient characteristics, as the reported cumulative incidence was not adjusted for any confounding effect such as comorbidities and medications. However, confounding variables might exist to bias the estimation. The proportion of patients with comorbidities in the decreasing group was higher compared to other groups [31]. This may indicate that higher comorbidity may mask the beneficial effect of HbA1c reduction on mortality. It highlighted the importance of adjusting for potential confounders to obtain an unbiased association between trajectories and mortality risk. Additionally, different HbA1c levels would also confound the association between the HbA1c trajectory and the risk of the outcome event. The results may be more valid if regression models with adjustment for comorbidities, medications, and HbA1c levels could be applied in the mentioned study [31].

The Australian study that classified patients into parallel trajectory groups also reported significant associations with risk of mortality [29]. However, the association varied among groups with different diabetes duration. A higher HbA1c trajectory (around 9%) was associated with a higher risk of mortality (hazard ratio (HR): 2.0, 95%CI: [1.1, 3.7]) for patients with a diabetes duration of less than five years, but a lower risk of mortality (HR: 0.56, 95%CI: [0.32, 0.96]) for patients with at least five years of diabetes duration compared with a lower HbA1c trajectory (around 6.5%). The findings in the short diabetes duration group concur with the other studies included in this review with comparable diabetes durations. The association observed in the long diabetes duration group suggests that hypoglycemia during the follow-up period might be associated with excess mortality, sometimes due to therapeutic intensification [44]. It could also be explained by the vascular, metabolic memory developed in patients with a longer diabetes duration, which means that prolonged hyperglycemia can reduce the benefit of subsequent glucose control. Therefore, established vascular changes and the heightened cardiovascular risk may not be reversed tight glycemic control [45].

Six out of the seven studies found that patients classified into the low, stable group were older than the other groups [28, 30–34]. This may be due to poorer glycemic control among young adults. Ali et al reported for 1,350 US adults that the age-standardized prevalence of poor glycemic control (HbA1c >9%) was different between adults aged 18–39 years (24.2%) and those aged ≥65 years (6.8%) [46]. Another study that also investigated glycemic control among different age groups found a lower risk of poor glycemic control (HbA1c >7.5%) in adults aged ≥60 years (adjusted odds ratio: 0.49, 95% confidence interval(CI): [0.28, 0.86]) compared with those aged <50 years [47]. Better glycemic control in older people might be explained by the differences in medications they were prescribed and education they received for optimal glycemic control [47].

### SBP

Opposing findings were observed between the association of SBP trajectories with risk of CVD and that with risk of mortality. An increasing SBP trajectory was associated with a higher risk of CVD but lower risk of mortality compared with a stable SBP (120–140 mmHg) trajectory. The effect of increasing SBP on CVD risk is consistent with the association between the burden of systolic HT (SBP>140mmHg) and increased risk of CVD, which has been well established in several previous studies [8, 48]. While the counterintuitive reduced risk of mortality associated with an increasing SBP trajectory could be due to the lack of adjustment for medications and comorbidities, which might have confounding effects on the SBP change and heightened mortality risk [36]. More analyses will need to be performed to explore the reasons for this association.

### eGFR

Both a low eGFR trajectory (35–45 mL/min/1.73m^2^) and a high/decreasing eGFR trajectory (from 90 to 70 mL/min/1.73m^2^) were associated with a higher risk of mortality than a medium eGFR trajectory (60–70 mL/min/1.73m^2^), which is consistent with the U-shaped association between eGFR and mortality in DM patients. The effect of a low eGFR level on the risk of mortality has been well established [39]. The effect of a high eGFR level, however, might be explained by the higher prevalence of hyperfiltration in the high/decreasing eGFR trajectory group (7%) than the medium trajectory group (0%) as hyperfiltration is associated with increased mortality [38, 49]. Identifying patients with low or high/decreasing eGFR trajectories should take priority in DM management [11].

### BMI and lipid studies

No studies on BMI or lipids trajectories were included in this review as we only included studies on adults. There are, however, several well-established studies reporting significant associations between longitudinal trends of BMI or lipids and CVD in adolescents or non-DM/HT population [23, 50, 51]. Attard et al analyzed BMI changes from adolescence (mean age: 16.9 years) to adulthood (mean age: 28.8 years) and found significant associations between BMI trajectories (based on four measurements over 12 years) and risk of CVD [22]. However, the study was conducted in an adolescent cohort, thus might not provide enough evidence on the association between an adult’s BMI trajectory and the risk of CVD or mortality. Dayimu et al found significantly different CVD risks among distinct trajectory groups of lipid profile, but the study was not conducted in DM/HT patients [23]. Future studies would be needed to investigate the association between BMI or lipid trajectories and CVD risk among adults with DM or HT.

Findings from this review suggest that regular monitoring on cardiometabolic risk factors would be helpful to predict the risk of CVD/mortality for patients, especially those diagnosed with DM. In clinical practice, more attention should be paid to not only patients with suboptimal levels, as defined by current clinical guidelines but also patients with unstable trends of cardiometabolic risk factors.

This review has some strengths. Firstly, this is the first systematic review of studies on the association between the trajectory of cardiometabolic risk factors and risk of CVD or mortality in DM or HT population. Moreover, it comprehensively reviewed all the trajectory analyses on five risk factors. Additionally, PRISMA guidelines were followed throughout the review to ensure good quality. There are also several limitations. Firstly, only qualitative comparisons among studies were conducted in this study since there is no established method that could quantitatively analyze results from trajectory analyses. Secondly, this review was restricted to English studies found in selected databases. Therefore, some studies published in local journals or other languages might be omitted. Lastly, only one out of the 11 included studies was conducted in a developing country, while all the other studies were conducted in developed high-income countries. This might weaken the generalizability of findings in this review as the risk of CVD or mortality and the overall level of glycemic control or blood pressure control might vary between developed counties and developing countries for discrepant health service level or education level.

## Conclusion

This review identified 11 studies that analyzed trajectory patterns for HbA1c, SBP, and eGFR and their association with risk of CVD or mortality in patients with DM or HT. The risk of CVD and mortality are associated with trajectories in most of the studies, with relatively stable and well-controlled trajectories being associated with the lowest risk of CVD and mortality. These findings suggest that a greater focus should be given to patients with both suboptimal control and unstable trends of cardiometabolic risk factors. Risk factor trajectories together with their single time point measurements have important clinical implications. More studies analyzing trajectories of cardiometabolic risk factors, including BMI and lipids, will be valuable to provide more comprehensive findings that could benefit patients with DM or HT.

## Supporting information

S1 Table Complete searching strategy in all databases. (PDF)

S2 Table Supplementary data collection table. (PDF)

S1 File Newcastle-Ottawa quality assessment form for cohort studies. (PDF)

S2 File PRISMA checklist. (DOCX)
