# Supplementary material for: The association between trajectories of risk factors and risk of cardiovascular disease or mortality among patients with diabetes or hypertension: A systematic review
Source: PLoS One. 2022 Jan 27;17(1):e0262885. doi: 10.1371/journal.pone.0262885 (PMC8794125; doi:10.1371/journal.pone.0262885)
Supplement: S1 Table — (PDF) [file pone.0262885.s001.pdf]

Supplementary table 1. Complete searching strategy in all databases

Database	Complete searching strategy
PubMed	("cardiovascular"[All Fields] OR "coronary disease*"[All Fields] OR "myocardial ischaemia"[All Fields] OR "coronary artery disease"[All Fields] OR "infarct*"[All Fields] OR "stroke*"[All Fields] OR "heart failure*"[All Fields] OR "death*"[All Fields] OR "mortalit*"[All Fields] OR "CVD"[All Fields] OR "chd"[All Fields] OR "die"[All Fields]) AND (("glycated hemoglobin a"[All Fields] OR "glycated haemoglobin a"[All Fields] OR "hba1c*"[All Fields] OR "haemoglobin a1c"[All Fields] OR "hemoglobin a1c"[All Fields] OR "glucose*"[All Fields]) OR ("bp"[All Fields] OR"blood pressure"[All Fields] OR "pulse pressure"[All Fields] OR "arterial pressure"[All Fields]) OR ("lipid*"[All Fields] OR "cholesterol*"[All Fields] OR "triglycerid*"[All Fields]) OR ("egfr"[All Fields] OR "glomerular filtration rate"[All Fields] OR "creatinin*"[All Fields]) OR ("BMI"[All Fields] OR "body mass index"[All Fields] OR "body weight"[All Fields] OR "obeses"[All Fields] OR "obesity"[All Fields] OR "obese"[All Fields] OR "obesities"[All Fields] OR "overweight*"[All Fields])) AND ("trajector*"[All Fields] OR "trend*"[All Fields] OR "longitudinal*"[All Fields] OR "long-term change*"[All Fields] OR "track*"[All Fields] OR "secular trend"[All Fields] OR "progression*"[All Fields] OR "latent class growth model*"[All Fields] OR "latent class growth mixture model*"[All Fields] OR "growth mixture model*"[All Fields] OR "latent growth model*"[All Fields] OR "latent class growth analysis"[All Fields] OR "latent class growth analyses"[All Fields] OR "group based trajectory model*"[All Fields] OR "group based trajectory analysis"[All Fields] OR "group based trajectory analyses"[All Fields] OR "group based model*"[All Fields] OR "latent growth mixture model*"[All Fields] OR "group based trajector*"[All Fields]) AND ("diabet*"[All Fields] OR "hypertensi*"[All Fields]) AND ((humans[Filter]) AND (english[Filter]) AND (alladult[Filter]))
MEDLINE	(cardiovascular.mp. OR coronary disease*.mp. OR myocardial ischaemia.mp. OR coronary artery disease.mp. OR infarct*.mp. OR stroke*.mp. OR heart failure*.mp. OR death*.mp. OR mortalit*.mp. OR CVD.mp. OR chd.mp. OR die.mp.) AND ((glycated hemoglobin a.mp. OR glycated haemoglobin a.mp. OR hba1c*.mp. OR haemoglobin a1c.mp. OR hemoglobin a1c.mp. OR glucose*.mp.) OR (bp.mp. OR blood pressure.mp. OR pulse pressure.mp. OR arterial pressure.mp.) OR (lipid*.mp. OR cholesterol*.mp. OR triglycerid*.mp.) OR (egfr.mp. OR glomerular filtration rate.mp. OR creatinin*.mp.) OR (BMI.mp. OR body mass index.mp. OR body weight.mp. OR obeses.mp. OR obesity.mp. OR obese.mp. OR obesities.mp. OR overweight*.mp.)) AND (trajector*.mp. OR trend*.mp. OR longitudinal*.mp. OR long-term change*.mp. OR track*.mp. OR secular trend.mp. OR progression*.mp. OR latent class growth model*.mp. OR latent class growth mixture model*.mp. OR growth mixture model*.mp. OR latent growth model*.mp. OR latent class growth analysis.mp. OR latent class growth analyses.mp. OR group based trajectory model*.mp. OR group based trajectory analysis.mp. OR group based trajectory analyses.mp. OR group based model*.mp. OR latent growth mixture model*.mp. OR group based trajector*.mp.) AND (diabet*.mp. OR hypertensi*.mp.)<br>Limit to (english language and humans and "all adult (19 plus years)")
Embase	(cardiovascular.mp. OR coronary disease*.mp. OR myocardial ischaemia.mp. OR coronary artery disease.mp. OR infarct*.mp. OR stroke*.mp. OR heart failure*.mp. OR death*.mp. OR mortalit*.mp. OR CVD.mp. OR chd.mp. OR die.mp.) AND ((glycated hemoglobin a.mp. OR glycated haemoglobin a.mp. OR hba1c*.mp. OR haemoglobin a1c.mp. OR hemoglobin a1c.mp. OR glucose*.mp.) OR (bp.mp. OR blood pressure.mp. OR pulse pressure.mp. OR arterial pressure.mp.) OR (lipid*.mp. OR cholesterol*.mp. OR triglycerid*.mp.) OR (egfr.mp. OR glomerular filtration rate.mp. OR creatinin*.mp.) OR (BMI.mp. OR body mass index.mp. OR body weight.mp. OR obeses.mp. OR obesity.mp. OR obese.mp. OR obesities.mp. OR overweight*.mp.)) AND (trajector*.mp. OR trend*.mp. OR longitudinal*.mp. OR long-term change*.mp. OR track*.mp. OR secular trend.mp. OR progression*.mp. OR latent class growth model*.mp. OR latent class growth mixture model*.mp. OR growth mixture model*.mp. OR latent growth model*.mp. OR latent class growth analysis.mp. OR latent class growth analyses.mp. OR group based trajectory model*.mp. OR group based trajectory analysis.mp. OR group based trajectory analyses.mp. OR group based model*.mp. OR latent growth mixture model*.mp. OR group based trajector*.mp.) AND (diabet*.mp. OR hypertensi*.mp.)<br>Limit to (human and english language and (article or article in press) and (adult <18 to 64 years> or aged <65+ years>))
