# Supplementary material for: The association between trajectories of risk factors and risk of cardiovascular disease or mortality among patients with diabetes or hypertension: A systematic review
Source: PLoS One. 2022 Jan 27;17(1):e0262885. doi: 10.1371/journal.pone.0262885 (PMC8794125; doi:10.1371/journal.pone.0262885)
Supplement: S2 Table — (PDF) [file pone.0262885.s002.pdf]

Supplementary table 2. Supplementary data collection table

Study (Author, Year of publication, Country/region)	Period of patient inclusion	Exposure period (for trajectories generation)	Outcome follow-up period	Criterion to measure goodness of fit of trajectory clustering model	Risk estimation adjusted for baseline or mean HbA1c value	Overall quality assessment result	Number of stars given in NOS selection item 1	Number of stars given in NOS selection item 2	Number of stars given in NOS selection item 3	Number of stars given in NOS selection item 4	Number of stars given in NOS comparability item	Number of stars given in NOS outcome item 1	Number of stars given in NOS outcome item 2	Number of stars given in NOS outcome item 3
Sridharan Raghavan et al, 2020, United States	2005.10-2016.09	Last measurement within 2 years before baseline to 2 years after baseline	Within 2 years after baseline	BIC; Mean posterior probability	No	Good	0	1	1	1	1	1	1	0
Beatriz Hemo et al, 2020, Israel	2004.01-2008.12	Within 5 years after baseline	6 years after baseline to 13 years after baseline	-	Yes	Good	1	1	1	1	1	1	1	0
Miyang Luo et al, 2017, Singapore	2004.11-2010.11	Within 5 years before baseline	Baseline to 2013.12 (myocardial infarction or stroke) / 2015.08 (death)	BIC; Mean posterior probability	Yes	Good	1	1	1	1	1	1	1	0
Hsing-Yi Chang et al, 2014, Taiwan	2003.08-2005.12	Within 4 years after baseline	Within 4.5 years after baseline	BIC	No	Good	1	1	1	1	1	1	1	0
T.M.E. Davis et al, 2016, Australia	1993.01-1996.12	Baseline to 5th annual review	5th annual review to 2012.12.31	BIC; Mean posterior probability; Minimal group size	No	Good	1	1	1	1	1	1	1	0
Tomas Karpati et al, 2018, Israel	2010.01	2007.01.01 to 2010.01.01	2010.01.01 to 2014.12.31	NbClust package in R	No	Good	1	1	1	1	1	1	1	0
Neda Laiteerapong et al, 2016, United States	1997.01-2001.12	Within 10 years after baseline	10 years after baseline to 2013.12.30	Lo-Mendell-Rubin fit index	Yes	Good	1	1	1	1	1	1	1	0
SanketS. Dhruva et al, 2017, United States	1994.02-1998.01	Within 6 months after baseline	6 months to 24 months after baseline	BIC; Mean posterior probability; Minimal group size	Yes	Good	1	0	1	1	1	1	1	0
Zhijun Wu et al, 2016, China	2006.01-2007.12	2006.01 to 2007.12	2008.01 to 2014.12	-	No	Good	1	1	0	1	1	1	1	0
Iris Walraven et al, 2015, Netherlands	1998.01-2011.12	Within 9 years after baseline	Within 9 years after baseline	BIC; Mean posterior probability; Usefulness and clinical interpretation	No	Good	1	1	1	1	1	1	1	0
Timothy M E Davis et al, 2016, Australia	1993.01-1996.12	Baseline to 5th annual review	5th annual review to 2012.12.31	BIC; Mean posterior probability; Minimal group size	No	Good	1	1	1	1	1	1	1	0

NOS = Newcastle-Ottawa quality assessment scale; BIC = Bayesian information criterion.
