# Supplementary material for: The association between trajectories of risk factors and risk of cardiovascular disease or mortality among patients with diabetes or hypertension: A systematic review
Source: PLoS One. 2022 Jan 27;17(1):e0262885. doi: 10.1371/journal.pone.0262885 (PMC8794125; doi:10.1371/journal.pone.0262885)
Supplement: S1 File — (PDF) [file pone.0262885.s003.pdf]

Newcastle-Ottawa Quality Assessment Form for Cohort Studies

Note: A study can be given a maximum of one star for each numbered item within the Selection and Outcome categories. A maximum of two stars can be given for Comparability.

Selection

1) Representativeness of the exposed cohort
    a) Truly representative ***(one star)***
    b) Somewhat representative ***(one star)***
    c) Selected group
    d) No description of the derivation of the cohort
2) Selection of the non-exposed cohort
    a) Drawn from the same community as the exposed cohort ***(one star)***
    b) Drawn from a different source
    c) No description of the derivation of the non exposed cohort
3) Ascertainment of exposure
    a) Secure record (e.g., surgical record) ***(one star)***
    b) Structured interview ***(one star)***
    c) Written self report
    d) No description
    e) Other
4) Demonstration that outcome of interest was not present at start of study
    a) Yes ***(one star)***
    b) No

Comparability

1) Comparability of cohorts on the basis of the design or analysis controlled for confounders
    a) The study controls for age, sex and marital status ***(one star)***
    b) Study controls for other factors (list) __________________________________ ***(one star)***
    c) Cohorts are not comparable on the basis of the design or analysis controlled for confounders

Outcome

1) Assessment of outcome
    a) Independent blind assessment ***(one star)***
    b) Record linkage ***(one star)***
    c) Self report
    d) No description
    e) Other
2) Was follow-up long enough for outcomes to occur
    a) Yes ***(one star)***
    b) No

Indicate the median duration of follow-up and a brief rationale for the assessment above:_____________________

3) Adequacy of follow-up of cohorts
    a) Complete follow up- all subject accounted for ***(one star)***
    b) Subjects lost to follow up unlikely to introduce bias- number lost less than or equal to 20% or description of those lost suggested no different from those followed. ***(one star)***
    c) Follow up rate less than 80% and no description of those lost
    d) No statement

Thresholds for converting the Newcastle-Ottawa scales to AHRQ standards (good, fair, and poor):

Good quality: 3 or 4 stars in selection domain AND 1 or 2 stars in comparability domain AND 2 or 3 stars in outcome/exposure domain

Fair quality: 2 stars in selection domain AND 1 or 2 stars in comparability domain AND 2 or 3 stars in outcome/exposure domain

Poor quality: 0 or 1 star in selection domain OR 0 stars in comparability domain OR 0 or 1 stars in outcome/exposure domain
